# TBK1 and IRF3 are potential therapeutic targets in Enterovirus A71-associated diseases

**DOI:** 10.1371/journal.pntd.0011001

**Published:** 2023-01-10

**Authors:** Wangquan Ji, Tiantian Sun, Dong Li, Shuaiyin Chen, Haiyan Yang, Yuefei Jin, Guangcai Duan

**Affiliations:** 1 Department of Epidemiology, College of Public Health, Zhengzhou University, Zhengzhou, China; 2 Henan Key Laboratory of Molecular Medicine, Zhengzhou University, Zhengzhou, China; University of Malaya Faculty of Medicine, MALAYSIA

## Abstract

**Background:**

Enterovirus A71 (EV-A71) is an important causative agent of hand-foot-and-mouth disease (HFMD) associated with enormous healthcare and socioeconomic burden. Although a range of studies about EV-A71 pathogenesis have been well described, the underlying molecular mechanism in terms of innate immune response is still not fully understood, especially the roles of TANK-binding kinase 1 (TBK1) and interferon-regulatory factor 3 (IRF3).

**Methodology/principal findings:**

Here, we applied TBK1 inhibitor and IRF3 agonist, for the first time, to evaluate the antiviral activities of TBK1 and IRF3 *in vivo*. We found that, through regulating EV-A71-induced type I interferon (IFN) response, IRF3 agonist effectively alleviated EV-A71-induced illness, while TBK1 inhibitor aggravated disease progression. In addition, EV-A71 replication was suppressed in EVA-71-infected mice administrated with IRF3 agonist. On the other hand, more severe pathological alterations of neuronal degeneration, muscle fiber breaks, fractured or fused alveolar walls, and diffuse congestion occurred in EVA-71-infected mice treated with TBK1 inhibitor administration. Furthermore, we determined the concentrations of interleukin (IL)-6, tumor necrosis factor-alpha (TNF-α), IL-1β, monocyte chemotactic protein-1 (MCP-1), and IL-10 in both lungs and brains of mice and found that TBK1 inhibitor promoted EV-A71-induced inflammatory response, while IRF3 agonist alleviated it, which was consistent with clinical manifestations and pathological alterations.

**Conclusions:**

Collectively, our findings suggest that TBK1 and IRF3 are potential therapeutic targets in EV-A71-induced illness.

## Author summary

EV-A71 is an important causative agent of HFMD. There is currently no specific antiviral agent to treat HFMD and the related severe complications. The underlying molecular mechanism controlling host innate immunity after EV-A71 infection is still not fully understood, especially the roles of TBK1 and IRF3. Here, we applied TBK1 inhibitor (TBK1/IKKε-IN-2) and IRF3 agonist (KIN1148) to evaluate the antiviral activities of TBK1 and IRF3 *in vivo*. We found through regulating EV-A71-induced type I IFN response, IRF3 agonist effectively alleviated EV-A71-induced illness, while TBK1 inhibitor aggravated disease progression. More severe pathological alterations of neuronal degeneration, muscle fiber breaks, fractured or fused alveolar walls and diffuse congestion occurred in EVA-71-infected mice with TBK1 inhibitor treatment, while no obvious pathological change occurred in EVA-71-infected mice with IRF3 agonist treatment and control mice. Furthermore, TBK1 inhibitor promoted EV-A71-induced inflammatory response, while IRF3 agonist alleviated it. Our findings suggest that TBK1 and IRF3 are potential therapeutic targets in EV-A71-induced illness for the development of new antiviral agents and therapeutic strategies.

## Introduction

The outbreak of hand-foot-and-mouth disease (HFMD) caused by human *Enteroviruses* (EVs) infection has become a global public health issue over the past few decades. Enterovirus A71 (EV-A71) is considered as the most important causative agent of HFMD, even although EV-A71 vaccines are available [1]. EV-A71 is a single-stranded, positive-sense RNA virus with approximately 7,500 nucleotides in length. A single open reading frame encodes a large single polyprotein precursor that is proteolytically cleaved into four structural (VP1, VP2, VP3, VP4) and seven non-structural (2A, 2B, 2C, 3A, 3C, and 3B, 3D) proteins in a proteolytic manner by the virus-encoded proteases 2Apro and 3Cpro [2]. The symptoms of most EV-A71 patients are mild and self-limiting, but some patients develop severe complications, such as aseptic meningitis, encephalitis, acute flaccid paralysis, and fatal cardiopulmonary failure [3]. Frequent outbreaks of HFMD in the Asia-Pacific region impose a huge burden on healthcare and socio-economics. There are still no specific antiviral agents against EV-A71 [4]. Accumulating evidence suggests that EV-A71 has evolved to use different strategies to evade the surveillance of host innate immune system via different proteases (e.g., 2Apro, 3Cpro, 2Cpro, and 3Dpro) [5,6]. Therefore, host innate immunity might be potential targets for effective treatment of EV-A71-associated HFMD. However, the molecular events governing this process are less well understood, making it necessary to further investigate the innate immune response during the pathogenesis of HFMD.

Innate immunity, the first line of host defense against invading pathogens, limits the virus replication and provides time for the followed adaptive immune response, which is necessary for the clearance of viral pathogen [7,8]. One of the most important antiviral processes is the recognition of pathogen-associated molecular patterns (PAMPs) by pattern recognition receptors (PRRs) [e.g., retinoic acid-inducible gene I (RIG-I)-like receptors (RLRs), Toll-like receptors (TLRs), and NOD-like receptors (NLRs)] to trigger innate immune response [9,10]. As reviewed in previous studies [5,6,11], the activation of several PRRs recruits various adaptors to directly interact with tumor necrosis factor receptor-associated factor 3 (TRAF3) [10,12]. In turn, TRAF3 interacts with the IκB kinase (IKK)-related kinases, such as TANK-binding kinase 1 (TBK1), which is different from other IKK-related kinases and indispensable in antiviral innate immunity [13]. The interferon regulatory factors (IRFs) are a family of transcription factors that play pivotal roles in many aspects of innate immune response and IRF3 is one of important regulators of type I interferons (IFNs) expression [14,15]. TBK1 is an important serine/threonine-protein kinase that mediates phosphorylation and nuclear translocation of IRF3, which contributes to induction of type I IFNs in the innate antiviral response [6]. Type I IFNs have diverse effects on innate and adaptive immune cells directly and/or indirectly through the induction of other mediators, which are critical for the control of many infections [16]. Type I IFNs induce the expression of hundreds of interferon-stimulated genes (ISGs) and eventually sustain an antiviral state [17]. Collectively, it is universally acknowledged that the innate immune response utilizes TBK1 to phosphorylate IRF3 and then trigger the transcription of type I IFNs. In other words, TBK1 and IRF3 are key molecules involved in innate immunity, whose activities are crucial for host defense responses against viruses. Nevertheless, multiple viruses have evolved sophisticated variety of strategies to facilitate their immune evasion by disrupting TBK1 and IRF3 activities to counteract and circumvent host antiviral response [18–20].

Both the precursors and mature proteins of EV-A71 play a dominant role in host innate immunity by cleaving and binding to different antiviral molecules [21–24]. EV-A71 3Cpro and 2Apro are the main antagonists of type I IFNs through cleaving major PRRs or key regulators of type I IFNs response [21]. For instance, the 3Cpro inhibits RIG-I-mediated IRF3 activation and type I IFN response [25]. The disruption of MDA5 by 2Apro diminishes the IRF3 activation and down-regulates type I IFN production [26]. The expression of sex-determining region Y-box 4 (Sox4) induced by EV-A71 attenuates the phosphorylation of TBK1 to repress the activation of TRAF3/TBK1 pathway [27]. TBK1 has a pivotal role in coordinating the activation of IRF3 in the innate immune response [19,28,29]. The IRF3 contributes to the production of type I IFNs that in turn amplifies the IFN response and the development of antiviral activity [30,31]. Although a range of studies about EV-A71 pathogenesis have been well described, the underlying molecular in terms of host innate immunity is still not fully understood, especially the participations of TBK1 and IRF3. Our previous study has demonstrated the involvement of IRF3 [32] and TBK1 during EV-A71 infection *in vitro*, but the roles of TBK1 and IRF3 *in vivo* have not been elucidated.

In present study, we applied TBK1 inhibitor (TBK1/IKKε-IN-2) and IRF3 agonist (KIN1148), for the first time, to evaluate the antiviral activities of TBK1 and IRF3 *in vivo*. Our findings suggest that TBK1 and IRF3 are potential therapeutic targets in EV-A71-induced illness, which will be beneficial for the development of new antiviral agents.

## Materials and methods

### Ethics statement

All experimental procedures were approved by the Life Science Ethics Review Committee of Zhengzhou University and carried out in accordance with the guidelines of Zhengzhou University for Animal Experiments (protocol code: ZZUIRB2020-29, date of approval: April 2019).

### Cell culture, viruses, and reagents

Vero cells (Africa green monkey kidney cells) were purchased from the National Collection of Authenticated Cell Cultures (Shanghai, China), which were cultured in Dulbecco’s modified Eagle’s medium (Thermo Fisher Scientific Inc., Waltham, MA, USA) supplemented with 2% or 10% fetal bovine serum (FBS), 100 U/mL of penicillin, 100 μg/mL of streptomycin, and incubated at 37 °C with 5% CO2. EV-A71 virus strain (ZZ1350, accession number: OP806304) was isolated from stool samples of a child with severe HFMD in Zhengzhou Children’s Hospital and conducted in Henan Provincial Center for Disease Control and Prevention, China [33]. To prepare viral stocks, viruses were propagated for one more passage in Vero cells. The titers were determined by a median tissue culture infective dose (TCID_50_) assay in accordance with the method of Reed and Muench [34]. Virus was harvested by three cycles of freeze-and -thaw and centrifuged at 4,000×g for 10 min at 4°C, filtered through a 0.22 μm filter, and stored at -80°C. In this study, the titer of EV-A71 stocks was 2.86 × 10^8^ TCID_50_/mL. KIN1148 (IRF3 agonist, Cat#: HY-101950) and TBK1/IKKε-IN-2 (TBK1 inhibitor, Cat#: HY-12453) were purchased from MCE (Med Chem Express, NJ, USA).

### Animals

Specific pathogen free (SPF) grade BALB/c mice were purchased from the Medical Animal Center in Zhengzhou University, Henan, China. Animals were raised in stainless steel cages in the Medical Animal Center located in the College of Public Health of Zhengzhou University on a 12 h light/dark cycle and allowed free access to food and water.

### Animal experiment

**Fig 1:**
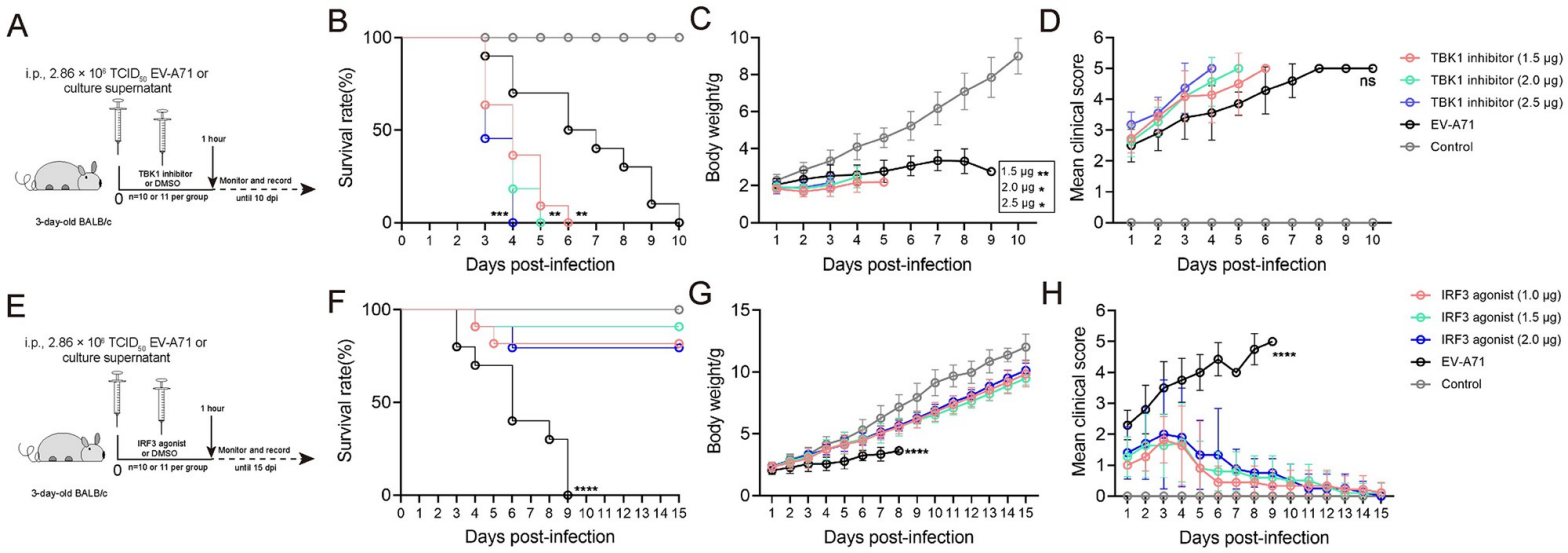
TBK1 and IRF3 interventions on EV-A71-induced disease severity in a neonatal mouse model. **(A**) and (**E**): The experimental design of TBK1 and IRF3 intervention. For TBK1 intervention, EV-A71 infected mice were treated with TBK1/IKKε-IN-2 (TBK1 inhibitor) via i.p. at a dose of 1.5, 2.0 or 2.5 μg per mouse at indicated time points. For IRF3 intervention, EV-A71 infected mice were treated with KIN1148 (IRF3 agonist) via i.p. at a dose of 1.0, 1.5 μg or 2.0 μg per mouse at indicated time points. Survival rates (**B**, **F**), body weight (**C**, **G**), and clinical scores (**D**, **H**) of mice were monitored and recorded daily up to 10 or 15 days after inoculation. Data represent the mean ± SD. TBK1 and IRF3 interventions mice with EV-A71 infection *vs* EV-A71-infected mice; *P < 0.05; **P < 0.01; *** *P* < 0.001; **** *P* < 0.0001; ns, no significance.

As described in our previous study [33], 3-day-old BALB/c mice were inoculated intraperitoneally (i.p.) with 2.86 × 10^6^ TCID_50_ EV-A71 (ZZ1350 strain, OP806304) to establish the infection model. In this study, 3-day-old BALB/c mice were i.p. inoculated with EV-A71, followed by i.p. injection with TBK1 inhibitor (dissolved in DMSO) to achieve a dose of 1.5, 2.0 or 2.5 μg per mouse [35] in a volume of 20 μL within 1 h (Three corresponding experimental groups, n = 10–11 per group) (Fig 1A), or followed by i.p. injection with IRF3 agonist (dissolved in DMSO) to achieve a dose of 1.0, 1.5 or 2.0 μg per mouse [36] in a volume of 20 μL within 1 h (Three corresponding experimental groups, n = 10–11 per group) (Fig 1E). The same volume of DMSO was given in the control group. After treatment, mice were monitored daily until 10 or 15 days post-infection (dpi) for clinical symptoms and survival rates. The specific experimental procedures of TBK1 and IRF3 interventions are shown in Fig 1A and E. Clinicals scores were defined as follows: 0, healthy; 1, reduced mobility; 2, ruffled hair, hunchbacked, or ataxia; 3, weight loss; 4, limb weakness and; 5, dying or death.

### Quantitative PCR (qPCR)

At 1 and 5 dpi, 6 group of mice (control, IRF3 agonist, TBK1 inhibitor, EV-A71, IRF3 agonist + EV-A71, TBK1 inhibitor + EV-A71; n = 3–4 per group) were euthanized and relevant tissues were harvested under aseptic conditions. In particular, mice in all groups showed no obvious clinical symptoms at 1 dpi. At 5 dpi, uninfected mice were all still healthy, while the infected mice showed different disease states in different groups but similar in the same group (See the detail clinical score in Fig 1). Total RNAs were extracted from homogenized tissues using TRIzol reagent (Thermo Fisher Scientific Inc., Waltham, MA, USA) and then reverse transcribed into cDNAs using a kit according to the supplier’s instruction (Yeasen Biotechnology Co., Ltd., Shanghai, China). qPCR analysis was done using the instrument (Serial No. q225-0207, Kubo Tech Co., Ltd., Beijing, China). The primers used in the present study were listed in Table 1. The data represent relative expression levels of target RNAs normalized to β-actin and calculated by the 2^–ΔΔCt^ method.

**Table 1:** Primers used in this study.

Gene	Forward	Reverse	Product Lengths (bp)
**VP1**	GCAGCCCAAAAGAACTTCAC	ATTTCAGCAGCTTGGAGTGC	226
**IFN-α**	CCACAGCCCAGAGAGTGACCAG	AGGCCCTCTTGTTCCCGAGGT	154
**IFN-β**	GCGTTCCTGCTGTGCTTCTCC	TGAAGTCCGCCCTGTAGGTGAG	145
**Mouse β-actin**	GTGCTATGTTGCTCTAGACTTCG	ATGCCACAGGATTCCATACC	174

### Viral loads

At 5 dpi, the tissue samples were aseptically removed, weighed, and stored at −80°C. Total RNA was extracted by using 1 mL TRIzol reagent. The genomic RNA of EV-A71 was quantitated by qPCR with a pair of primers described in Table 1. The fragment (226 bp) amplified by the VP1 primers was cloned into the pET-28a (+) plasmid, producing a plasmid pET-28a (+)-EV-A71 VP1 that was used as a standard for quantification of EV-A71 copy numbers. A standard curve was generated from serially diluted pET-28a (+)-EV-A71 VP1 by linear regression: Y = −0.2468X + 12.397, R^2^ = 0.99. X represents CT value and Y represents Log_10_ (copy number). Briefly, our results were obtained based on the equations, CT values, and sample weights.The viral loads were calculated and expressed as Log_10_ (viral RNA copies)/mg tissues.

### Histopathology

At 5 dpi, the brains, spinal cords, lungs, hearts, and muscles were harvested from euthanized mice, washed by PBS, and fixed by immersion in 4% paraformaldehyde for 48 h at room temperature. The fixed tissue samples were then embedded in paraffin and sectioned (5 μm), followed by haematoxylin and eosin (H&E) staining. The protein expression of VP1 was determined by immunohistochemical (IHC) staining using a standard avidin-biotin immunoperoxidase technique as described previously [37]. All pictures were captured using an inverted fluorescence microscope (OLYMPUS, IX73). The VP1 antibody used in this study was purchased from GeneTex, Inc. (Cat#: GTX132339). Pathological score for each section was evaluated on scale of 0 to 4 with increments of 0.5 by three blinded observer for pathological lesion (H&E) [33].

### Cytokines

At 5 dpi, the concentrations of interleukin (IL)-6, tumor necrosis factor-alpha (TNF-α), IL-1β, monocyte chemotactic protein-1 (MCP-1) and IL-10 in the tissue lysates of brains and lungs were determined by enzyme-linked immunosorbent assay (ELISA) kits (Biolegend, San Diego, CA, USA) according to the manufacturer’s instructions. The concentration of total proteins was determined using a BCA protein assay kit (Beijing Biomed Gene Technology Co., Ltd., Beijing, China). The results were normalized by the concentration of total proteins in each tissue sample.

### Statistical analysis

All experiments were repeated at least three times. All statistical analysis was performed with GraphPad Prism Version 8.3 (GraphPad 8.3 Software, San Diego, CA, USA). The differences of survival rates in treated versus EV-A71 mice were assessed with the *Mantel-Cox log rank test* and survival curves were plotted using the *Kaplan-Meier method*. The results were expressed as the mean ± standard deviation (SD) or median with range (non-normal distribution). Statistical analysis was carried out using two-tailed *unpaired Student’s t test* or *Mann-Whiteny test* (non-normal distribution) for two groups. Differences between groups were considered as significance when the *P* value was less than 0.05.

## Results

### TBK1 and IRF3 interventions on EV-A71-induced disease severity in a neonatal mouse model

To evaluate the roles of TBK1 and IRF3 in EV-A71 infection, a TBK1 inhibitor and an IRF3 agonist were used in this study. We have conducted several preliminary experiments (S1A Fig) to define the adequate dose of TBK1 inhibitor in present study. IRF3 agonist treatment was dose-dependent with phosphorylated IRF3 *in vitro* (S1B Fig). The results from *in vitro* experiments showed that the TBK1 inhibitor had no obvious cytotoxicity (S2A Fig). In addition, the agonist/inhibitor did not directly affect the infectivity of virus (S2B Fig). We also tested the optimal intervention time point (S3 and S4 Figs) and finally chose to administer intervention within 1 hour after infection. EV-A71-infected mice displayed weight loss, reduced activity, hunchback, and limb paralysis, and died at 3–10 dpi (Fig 1B–1D and 1F–1H). EV-A71-infected mice with TBK1 inhibitor intervention were died at 3–6 dpi, which approximately 4 days earlier than EV-A71-infected mice (Fig 1B). However, there was no statistical difference in clinical scores (Fig 1D). In contrast, IRF3 agonist intervention significantly improved the survival rate of EV-A71-infected mice (Fig 1F) (*P* < 0.0001). In addition, differences with statistical significance in body weight (Fig 1G) and clinical scores (Fig 1H) were observed between the two groups. Almost all EV-A71-infected mice died within 9 dpi, while most IRF3-treated mice survived and their clinical scores began to decline at 5–6 dpi (Fig 1F and 1H). Based on these results, IRF3 agonist effectively alleviated EV-A71-induced illness, while TBK1 inhibitor worsened disease progression *in vivo*.

### TBK1 and IRF3 interventions on EV-A71-induced type I IFN response

**Fig 2:**
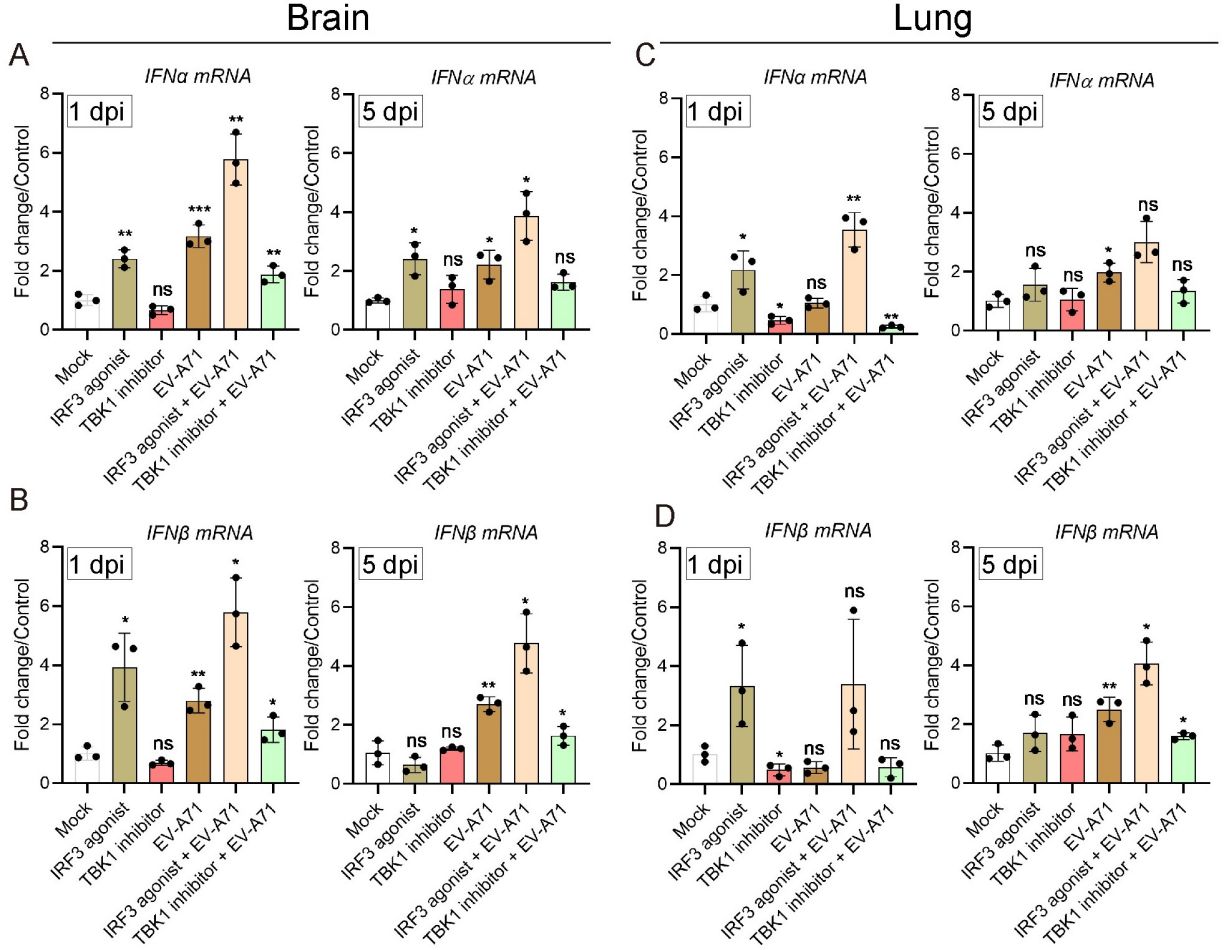
TBK1 and IRF3 interventions on type I IFN response in the brains and lungs of EV-A71-infected mice. qPCR was conducted to measure the expression levels of IFN-α and IFN-β in the brains (**A and B**) and lungs (**C and D**) of EV-A71-infected mice or mock mice with TBK1 and IRF3 interventions at 1 and 5 dpi. Results were normalized by β-actin. Data represent mean ± SD. n = 3 per group; TBK1 and IRF3 interventions alone, EV-A71 *vs* Mock; TBK1 and IRF3 interventions mice with EV-A71 infection *vs* EV-A71-infected mice; *P < 0.05; **P < 0.01; *** *P* < 0.001; ns, no significant. All the experiments were repeated at least three times.

To investigate whether TBK1 and IRF3 interventions affect type I IFN response, EV-A71-infected mice were treated with IRF3 agonist (2.0 μg per mouse) and TBK1 inhibitor (2.5 μg per mouse), and the mRNA levels of IFN-α and IFN-β in brain and lung tissues were detected by qPCR. At the early stage of intervention (1 dpi), IRF3 agonist could significantly increase the transcription level of IFN-α, and IFN-β in control mice and EV-A71-infected mice, while TBK1 inhibitor had a significant inhibitory effect (Fig 2). At 5 dpi, there was almost no change in the transcription levels of type- I IFNs in mice treated with drug alone (Fig 2). However, the TBK1 inhibitor significantly suppressed the transcription level of IFN-β in the brains (Fig 2B) and lungs (Fig 2D) of EV-A71-infected mice with about 2 folds (*P*<0.05). In addition, the TBK1 inhibitor also repressed the transcription level of IFN-α in brains (Fig 2A) and lungs (Fig 2C) of EV-A71 infected mcie, but the differences showed no statistical significance. In contrast, the IRF3 agonist significantly increased IFN-β mRNA expression in the brains (Fig 2B) and lungs (Fig 2D) of infected mice (*P*<0.05). In addition, the IRF3 agonist also enhanced IFN-α mRNA expression in brains (Fig 2A) and lungs (Fig 2C), but the difference showed no statistical significance in lungs. These results indicated that TBK1 and IRF3 interventions affected EV-A71-induced type I IFN response in mice.

### TBK1 and IRF3 interventions on the antiviral activity against EV-A71 in mice

**Fig 3:**
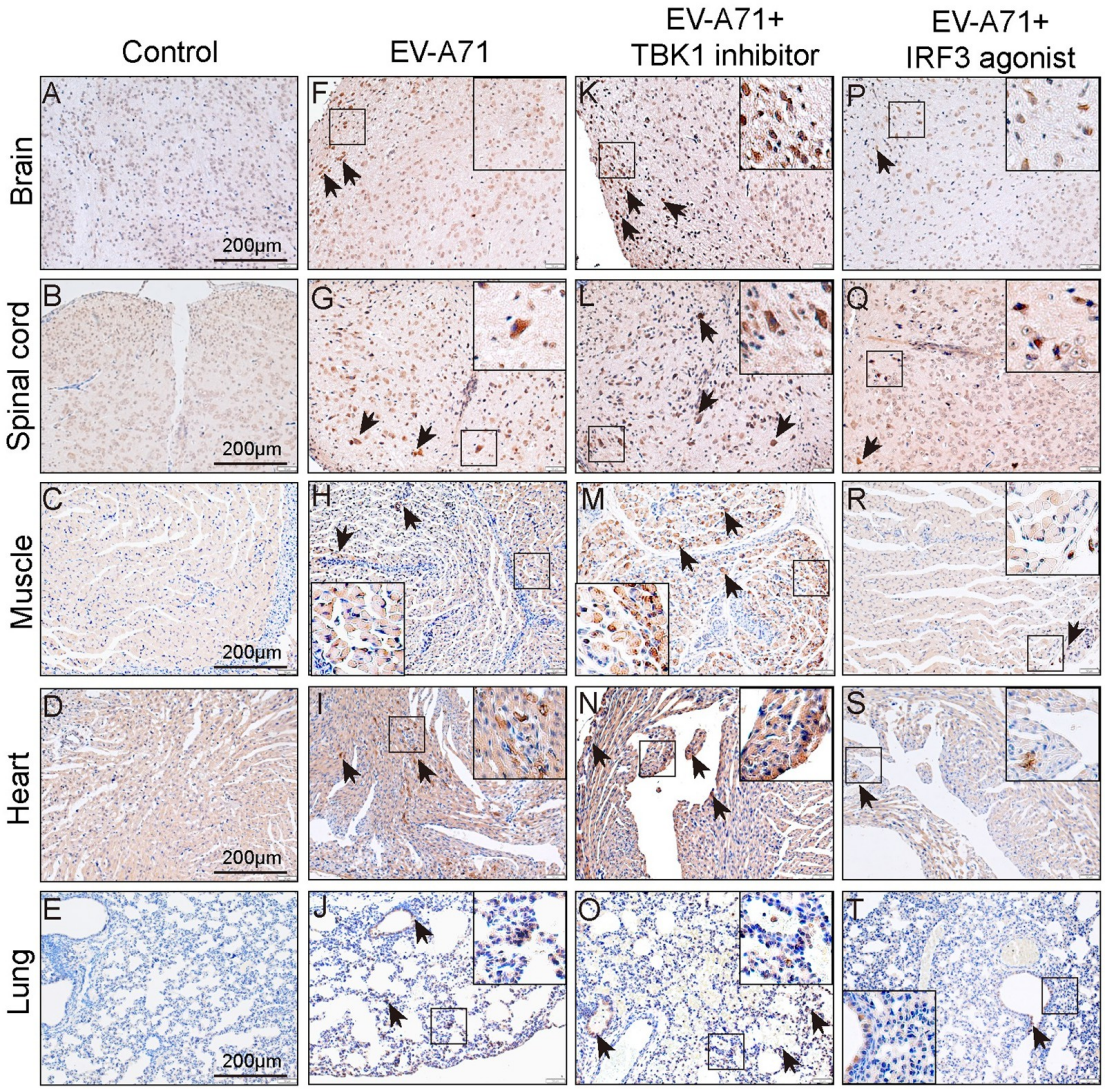
TBK1 and IRF3 interventions on the antiviral activity against EV-A71 in mice. The brains (**A, F, K, P**), spinal cords (**B, G, L, Q**), muscles (**C, H, M, R**), hearts (**D, I, N, S**) and lungs (**E, J, O, T**) of mice were dissected for immunohistochemical analysis at 5 dpi. The black arrows indicated the positive staining (brown) and pictures were captured using an inverted fluorescence microscope. Bar = 200 μm. n = 4 per group, all experiments were repeated three times.

**Fig 4:**
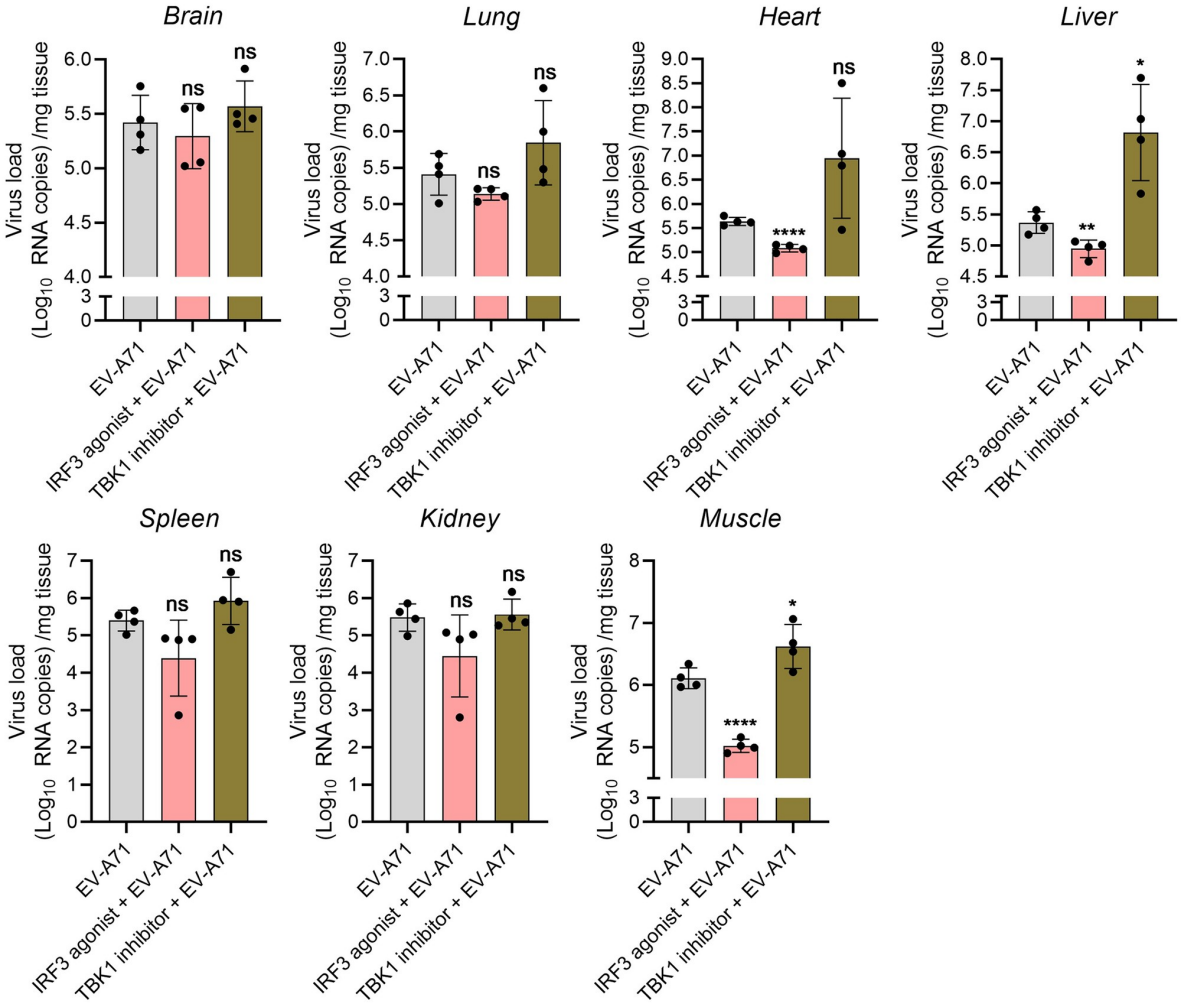
TBK1 and IRF3 interventions on EV-A71 replication. Viral loads in the brains, lungs, hearts, livers, kidneys, spleens and skeletal muscles of mice at 5 dpi were determined by qPCR. Results were expressed as Log_10_ RNA copies/mg tissue. n = 4 per group; TBK1 and IRF3 interventions in mice with EV-A71 infection *vs* EV-A71-infected mice; * *P* < 0.05; **P <0.01; **** *P* < 0.0001; ns, no significant. All the experiments were repeated at least three times.

In *vitro*, our studies showed that the phosphorylation of TBK1 and IRF3 in RD cells increased at the early stage and then decreased later after EV-A71 infection (S5A Fig), suggesting that the antiviral response was weakened. Overexpression of IRF3 had an antiviral effect similar to IRF3 agonist (S5B Fig). In contrast, siTBK1 or TBK1 inhibitors increased viral proliferation in RD cells (S5C Fig). To further understand the antiviral effects of TBK1 and IRF3 interventions *in vivo*, viral loads and VP1 expression were examined in relevant tissues. As shown in Fig 3, we didn’t detect any VP1 protein in the brains (Fig 3A), spinal cords (Fig 3B), muscles (Fig 3C), hearts (Fig 3D) and lungs (Fig 3E) of control mice. VP1 antigens were detected in above organs or tissues of EV-A71 infected mice (Fig 3F–3J). After treatment with TBK1 inhibitor, the positive area of VP1 antigens was significantly enhanced (Fig 3K–3O), which was contrary to the IRF3 agonist group (Fig 3P–3T). Next, we further measured the viral loads of brains, lungs, hearts, livers, kidneys, spleens and muscles using qPCR. The IRF3 agonist treatment enhanced the ability to fight against invading EV-A71 and partially overwhelmed EV-A71 replication (Fig 4). Notably, the viral loads in hearts, livers, and muscles were significantly decreased by the agonist compared with EV-A71 group (Fig 4). In contrast, the treatment of TBK1/IKKε-IN-2 (2.5 μg per mouse) significantly increased the viral loads in the liver and muscle tissues of EV-A71-infected mice (Fig 4). The viral loads of other tissues were also elevated without significant differences (Fig 4). In summary, these data suggested that TBK1 and IRF3 were potential targets for the development of antiviral drugs.

### TBK1 and IRF3 interventions on pathological changes in mice caused by EV-A71 infection

**Fig 5:**
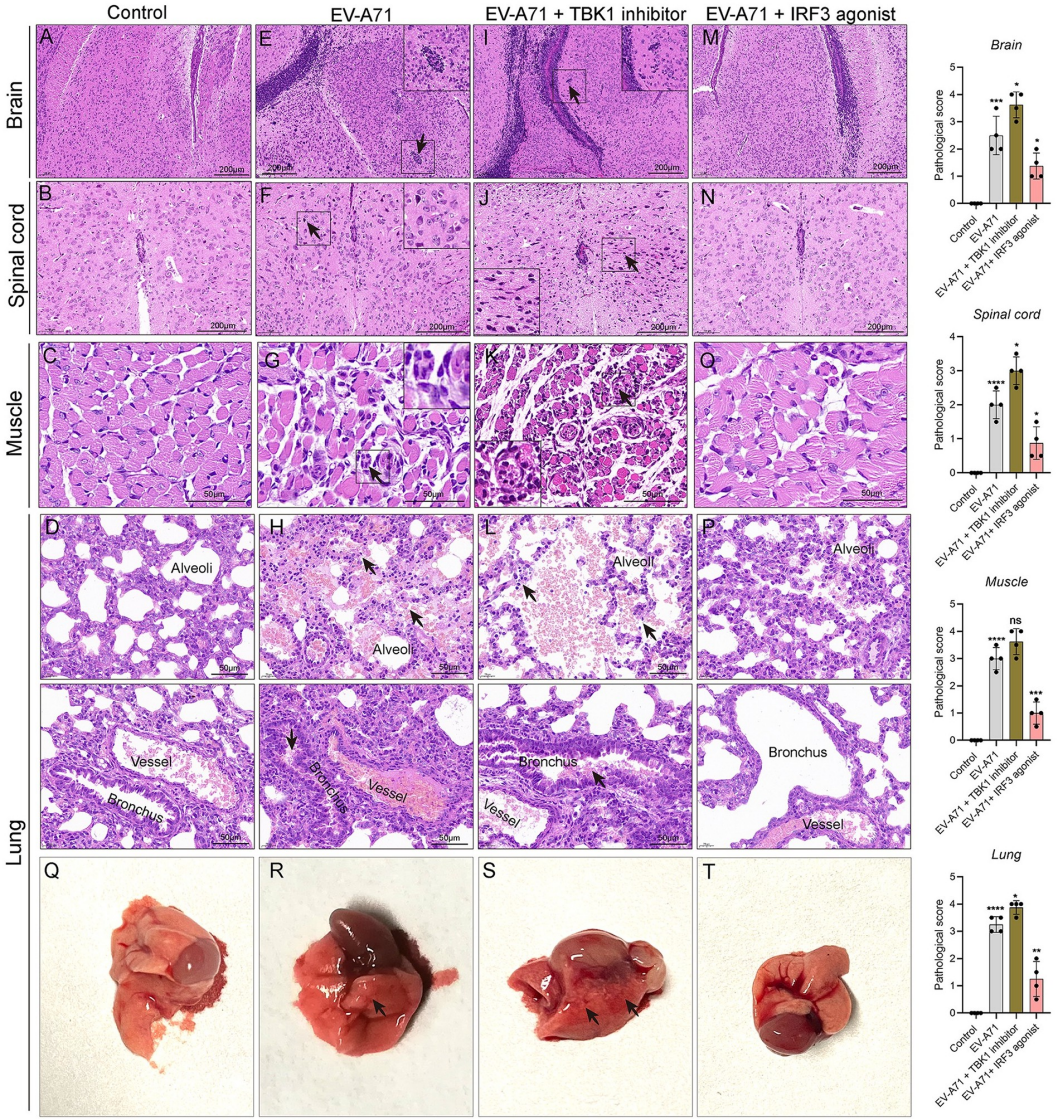
Pathological examination of mouse tissues. Pathological changes in the brains (**A, E, I, M**), spinal cords (**B, F, J, N**), muscles (**C, G, K, O**) and lungs (**D, H, L, P**) of mice at 5 dpi were evaluated by H&E staining. Scoring for each section was evaluated on scale of 0 to 4 with increments of 0.5 independently by three observers for pathological changes. n = 4 per group; EV-A71 *vs* Control; EV-A71-infected mice with TBK1 and IRF3 interventions *vs* EV-A71-infected mice; *P < 0.05; ** *P* < 0.01; *** *P* < 0.001; **** *P* < 0.0001; ns, not significant. (**Q-T**) Fresh lung tissues dissected from different experimental groups. Black arrows indicate typical pathological lesions. All experiments were repeated three times.

The brains, spinal cords, skeletal muscles, and lungs of mice from four groups (control mice, EV-A71-infected mice, EV-A71-infected mice with TBK1 inhibitor treatment, or EV-A71-infected mice with IRF3 agonist treatment) were sampled at 5 dpi and histologically examined by H&E staining (Fig 5A–5P). No obvious abnormality was observed in control mice (Fig 5A–5D). Neuronal degeneration and neuronophagia were found in the brains and spinal cords of the EV-A71-infected mice (Fig 5E and 5F). More severe damage with decreased number of neurons and neuronal degeneration occurred in the TBK1 inhibitor-treated mice (Fig 5I and 5J), whereas no obvious pathological changes occurred in IRF3 agonist-treated mice (Fig 5M and 5N). The skeletal muscle tissues were seriously damaged by EV-A71, displaying inflammatory infiltration and partial necrotic myositis (Fig 5G). More severe damage with diffuse lesions and muscle fiber breaks occurred in the TBK1 inhibitor-treated mice (Fig 5K), whereas no such pathological changes were observed in IRF3 agonist-treated mice (Fig 5O). The lung tissues were also affected by EV-A71, exhibiting focal congestion, edema (Fig 5H). Compared to EV-A71-infected mice, fractured or fused alveolar walls and diffuse congestion were observed in the TBK1 inhibitor-treated mice (Fig 5L), whereas the architecture of the lungs in IRF3 agonist-treated mice was minimally affected (Fig 5P). The pathological scores of the above tissues were consistent with the observation. Compared with the EV-A71 group, the TBK1 inhibitor treatment increased the pathological scores, while the IRF3 agonist treatment decreased the pathological scores. As shown in Fig 5Q–5T, the lungs of control and IRF3 groups showed normal pink color (Fig 5Q and 5T), but it looked reddish-brown and edema in EV-A71 group (Fig 5R), particularly the TBK1 inhibitor-treated group (Fig 5S). Our results suggested that TBK1 and IRF3 interventions affected EV-A71-induced pathological changes.

### TBK1 and IRF3 interventions on pro-inflammatory cytokine expression in EV-A71-infected mice

**Fig 6:**
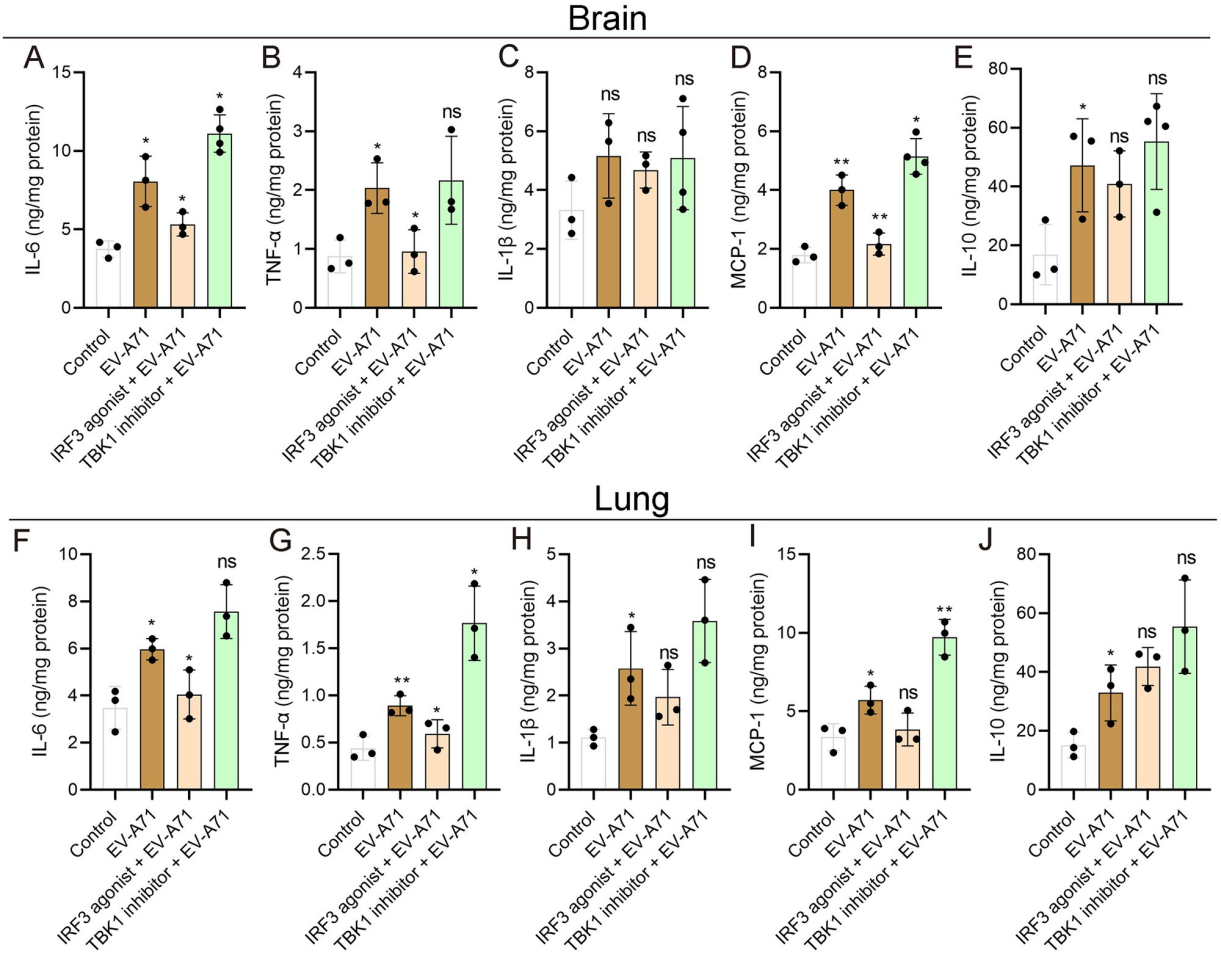
TBK1 and IRF3 interventions on EV-A71-induced proinflammatory cytokines expression in mice. The concentrations of IL-6, TNF-α, IL-1β, MCP-1and IL-10 in the brains (**A-E**) and lungs (**F-J**) of mice at 5 dpi were determined using ELISA. The data are shown as means ± SD. n = 3 or 4 per group; EV-A71 *vs* Control; EV-A71-infected mice with TBK1 and IRF3 interventions *vs* EV-A71-infected mice. * *P* < 0.05; **P < 0.01; ns, not significant. All the experiments were repeated at least three times.

The severity of EV-A71 infection was presumed to be caused by excessive and robust pro-inflammatory cytokine production. We further evaluated the effects of TBK1 and IRF3 interventions on EV-A71-induced proinflammatory cytokine expression. In the early stage (1 dpi), the transcription levels of TNF-α, MCP-1 and IL-6 were almost unchanged in mock mice after interventions (S6 Fig), so the drugs alone couldn’t cause drastic changes in cytokines production at the doses we used. Our data showed that the concentrations of IL-6 (Fig 6A and 6F), TNF-α (Fig 6B and 6G), MCP-1 (Fig 6D and 6I), and IL-10 (Fig 6E and 6J) were significantly increased in both brains (Fig 6A–6E) and lungs (Fig 6F–6J) of EV-A71-infected mice compared to control mice. In addition, the concentration of IL-1β was also increased in brains (Fig 6C, without statistical difference) and lungs (Fig 6H, *P<0*.*05*). Our results also indicated that the IRF3 agonist almost restrained the expressions of cytokines and almost reversed the effect of EV-A71 infection on cytokines production. The concentrations of IL-6 and TNF-α were significantly decreased in both brains and lungs with statistical difference (*P<0*.*05*). Although it was not statistically significant, the IRF3 agonist reduced the production of IL-1β and IL-10 in both brains (Fig 6C and 6E) and lungs (Fig 6H and 6J) compared with the EV-A71 group. Nevertheless, TBK1 inhibitor administration intensified cytokines production in EV-A71-infected mice. In both brains and lungs, the concentrations of IL-6 (Fig 6A and 6F), TNF-α (Fig 6B and 6G), IL-1β (Fig 6C and 6H), MCP-1(Fig 6D and 6I) and IL-10 (Fig 6E and 6J) were all elevated after TBK1 inhibitor treatment, but the differences of IL-1β (Fig 6C and 6H) and IL-10 (Fig 6E and 6J) were not statistically significant. To a certain extent, our data indicated that TBK1 inhibitor enhanced EV-A71-induced inflammatory response, while IRF3 agonist inhibited EV-A71-induced inflammatory response in mice. These results were consistent with the clinical manifestations and histopathological results.

## Discussion

Studying the molecular mechanisms of EV-A71-induced innate immunity is essential for understanding the pathogenic mechanism and developing effective antiviral drugs. In present study, activation of type I IFN response by administering an IRF3 agonist reduced viral loads and excessive production of cytokines and alleviated pathological damage caused by EV-A71, resulting in the prolonged survival of EV-A71-infected mice. In contrast, after treatment with a TBK1 inhibitor, the infected mice developed severe symptoms rapidly and died. Our data highlighted the important roles of TBK1 and IRF3 in innate antiviral response against EV-A71 and provided targets for the development of antiviral drugs.

Effective innate immune activation is essential for controlling viral infections. We first utilized a mouse model to evaluate the roles of TBK1 and IRF3 during EV-A71 infection. IRF3 is a well-characterized signaling mediator/transcription factor that is essential for activation of the RLR pathway. Activated IRF3 dimerizes and enters the nucleus to regulate both type I IFN and IFN-stimulated genes [38]. As expected, our data demonstrated that enhanced IRF3 activation improved the survival of infected mice and alleviated the symptoms of the disease caused by EV-A71. An IRF3 agonist, KIN1148, induced dose-dependent IRF3 nuclear translocation and specific activation of IRF3-responsive promoters [36]. On the other hand, mice treated with a TBK1 inhibitor died approximately 4 days earlier than untreated mice after EV-A71 infection, suggesting that TBK1 inhibitor contributed to EV-A71 pathogenesis. TBK1 inhibitor (TBK1/IKKε-IN-2) can inhibit TBK1 biochemical function and phosphorylation of IRF3 [35]. Therefore, suppression of TBK1 may prevent IRF3 phosphorylation and subsequently cause reduction of IFN-α/β production. Next, we investigated the transcription levels of IFN-α and IFN-β to verify the change of type I IFN production. We found that IRF3 agonist treatment increased EV-A71-induced IFN-α and IFN-β expression, while TBK1 inhibitor suppressed EV-A71-induced IFN-α and IFN-β expression.

Administration of type I IFNs can limit virus spread at an early phase. EV-A71 did not effectively stimulate production of type I IFNs in mice. Treatment wiht type I IFN could improve and even cure EV-A71 patients [6]. EV-A71 strains with different replication capacity are likely associated with EV-A71 pathogenesis [39]. HFMD severity is positively correlated with viral loads of EV-A71 in throat swabs [40]. The attenuation of virus loads was observed in the multiple organs of EV-A71-infected mice with IRF3 agonist treatment, which could be explained by the increased production of IFN-α/β. By contrast, treatment with a TBK1 inhibitor increased the viral loads in brain and lung tissues of EV-A71-infected mice. Accumulating evidence indicates that EV-A71 can directly infect neurons in the central nervous system (CNS) [41,42]. IFN-β expression was upregulated in EV-A71-infected neural cells via pattern recognition receptors (PRRs) sensing of virus RNA [43]. We believed that the viral proliferation was largely eliminated by the increased type I IFNs resulting from strong activation of IRF3/IFNs signal. TBK1 is necessary for IRF3 phosphorylation [29], so inhibition of TBK1 suppresses the downstream signals, resulting in type I IFNs reduction. A previous study reported that recombinant IFN-α could protect against lethal EV-A71 infection. In contrast, administration of neutralizing antibody against IFN-α/β resulted in the increased viral loads and disease severity [44]. A single dose of recombinant adenovirus expressing mouse IFN-α protected mice from lethal EV-A71 challenge [45]. Recombinant human IFN-α1b therapy reduced the fever clearance time, healing time of typical skin or oral mucosa lesions, and EV-A71 viral loads in children with HFMD [46]. Besides, many studies have described other strategies to stimulatr type I IFN production during EV-A71 infection [47–49]. These findings imply that the sequelae and mortality may be reduced if the production of type I IFNs can be normally induced during EV-A71 infection [47–49]. To further evaluate the effects of type I IFN activation, we performed pathological examinations in major target organs. We observed that IRF3 agonist alleviated pathological damage of EV-A71-infected mice. Severe neurological complications could lead to life threatening neurogenic pulmonary edema [3]. Our data indicated that IRF3 agonist reduced neuronal damage in the brains and spinal cords and alleviated muscle and lung pathologies, while TBK1 inhibitor exacerbated pathological changes after infection.

Uncontrolled inflammatory responses are considered to be associated with HFMD severity [50–52]. Excessive proinflammatory cytokine (e.g. TNF-α, IL-1β, IL-6, IL-10, and MCP-1) production plays an important role in the development of EV-A71-associated disease severity [52,53]. In present study, TBK1 inhibitor enhanced the expression of TNF-α, IL-1β, IL-6, IL-10, and MCP-1 in both brains and lungs after EV-A71 infection, but IRF3 agonist decreased the production of TNF-α, IL-6, and MCP-1 in both brains and lungs after EV-A71 infection, which were consistent with the clinical symptoms mentioned above. Cytokines vary in different severity of EV-A71 patients and serum concentrations of IL-1β were raised significantly in patients who developed cardio-respiratory compromise, suggesting a potential biomarker for predicting HFMD severity [54,55]. Higher plasma level of IL-10 is evident in critical than severe cases and IL-10-592C allele is associated with IL-10 expression and the severity of EV-A71 patients [50,56]. The levels of IL-6 and TNF-α in severe patients were significantly higher than that in mild patients [57–60]. MCP-1 in the brain appears to play an important role in elicitation of the response to encephalitis [61,62]. In the early stage (1 dpi), the transcription levels of TNF-α, MCP-1 and IL-6 were almost unchanged in mock mice after intervention. Therefore, alternations of inflammatory cytokine production might result from viral replication, but not IRF3/TBK1 treatment. Our findings suggested that excessive inflammatory response might be suppressed by activation of type I IFN signaling.

It is an inevitable lengthy and expensive process to discover and develop any given drug. Taken the imprecise and too distant from the human condition of animal model, it is difficult to determine how a drug acts and why it works, as well as to generate conclusive data to demonstrate the effectiveness in humans. In this study, many TBK1-treated mice died before 5 dpi, so the surviving mice were likely to have milder symptoms, which may underestimate the effect of this inhibitor. Therefore, animal models and the associated preclinical data are not inherently infallible, which will make failed translation from preclinical animal model-based studies to clinical evaluations. For evaluating the safety/toxicity and determining the appropriate drug dose, it still needs to be investigated in human subjects.

## Conclusion

Our findings suggest that TBK1 and IRF3 are potential therapeutic targets in EV-A71-associated diseases, which will be beneficial for the development of new antiviral agents and therapeutic strategies.

## Supporting information

S1 Fig Administration of TBK1 inhibitor (TBK1/IKKε-IN-2) on mice survival at different doses and the effect of IRF3 agonist in *vitro*. (A) 3-day-old BALB/c mice were inoculated intraperitoneally (i.p.) with 2.86 × 10^6^ TCID_50_ EV-A71 (ZZ1350 strain, OP806304), followed by i.p. injection with TBK1 inhibitor (dissolved in DMSO) to achieve a dose of 0.1–5.0 μg per mouse within 1 hour post infection (hpi). After being administered different doses of TBK1 inhibitor, survival rates of infected and control mice were recorded until 15 dpi. Finally, considering the survival rate, economic and reproducibility, 2.5 μg per mouse was selected as the appropriate intervention dose for following experiments. Statistical differences of survival rates between control and treated mice were assessed with the *Mantel-Cox log rank test* and survival curves were plotted using the *Kaplan-Meier method*. n = 10 per group; * *P*< 0.05; ** *P*< 0.01; *** *P*< 0.001; **** *P*< 0.0001. (B) IRF3 agonists treatment was dose-dependent with phosphorylated IRF3 (pIRF3) in *vitro*.
(TIF)

S2 Fig Cytotoxicity of TBK1 inhibitor (TBK1/IKKε-IN-2) at different concentrations and the interaction effect between the virus and agonist/inhibitor. (A) 10^4^ RD cells were seeded to each well of the 96-well plate and cultured for 24 h. Then, cells were cultured with DMSO or TBK1 inhibitor (0–10 μM) for 24 h before performing CCK8 assays (Biosharp, Cat#: BS350B)) according to the supplier’s instructions. n = 6 wells/per group (B) The agonist/inhibitor are not directly viricidal. The virus (2.86 × 10^8^ TCID_50_/mL) with or without agonist/inhibitor (100μM) was mixed and incubated for 1h at 37°C. We incubated the virus or the virus-agent mixture (MOI = 1) with monolayer RD cells for 1h at 4 °C (n = 4 wells/per group). After removing the culture medium, the cells were washed three times with cold PBS to remove the unbound viral particles. At 12 hpi, we freeze-thawed RD cells three times to assay the TCID_50_ of RD cell lysates and quantify the effect of the agonist/inhibitor on EV-A71 infectivity. Results were normalized by the infectivity of the original EV-A71. Statistical analysis was carried out using two-tailed *unpaired Student’s t test*. data represent the mean ± SD, ns, no significant.
(TIF)

S3 Fig Administration of IRF3 agonist (KIN1148) on mice survival at different time points. 3-day-old BALB/c mice were inoculated i.p. with 2.86 × 10^6^ TCID_50_ EV-A71 to infected the mice, followed by i.p. injection with 2.0 μg IRF3 agonist per mouse (dissolved in DMSO) at different time points (1, 6, 12, 24, 48, 72 hpi). Survival rates of infected and control mice were recorded until 15 dpi. Statistical differences of survival rates between control and treated mice were assessed with the *Mantel-Cox log rank test* and survival curves were plotted using the *Kaplan-Meier method*. n = 10 per group; ** *P*< 0.01; *** *P*< 0.001; **** *P*< 0.0001.
(TIF)

S4 Fig Administration of TBK1 inhibitor (TBK1/IKKε-IN-2) on mice survival at different time points. 3-day-old BALB/c mice were inoculated i.p. with 2.86 × 10^6^ TCID_50_ EV-A71, followed by i.p. injection with TBK1 inhibitor (dissolved in DMSO) to achieve a dose of 2.5 μg per mouse at different time points (1, 6, 12, 24, 48, 72 hpi). Survival rates of infected and control mice were record until 15 dpi. Statistical differences of survival rates between control and treated mice were assessed with the *Mantel-Cox log rank* test and survival curves were plotted using the *Kaplan-Meier* method. n = 10 per group; * *P*< 0.05; ** *P*< 0.01.
(TIF)

S5 Fig IRF3 and TBK1 are related to EV-A71 replication. (A) Changes of phosphorylated TBK1 and IRF3 after EV-A71 infection, MOI = 1. (B) Overexpression of IRF3 (Synthesized by Shenzhen BGI Co., LTD) and IRF3 agonist (100 nmol/L) could decrease the VP1 expression of EV-A71. (C) siTBK1 (Guangzhou RiboBio Co., Ltd.) or TBK1 inhibitors (100 nmol/L) could increase the viral proliferation. The expression of related proteins was detected by WB analysis.
(TIF)

S6 Fig In the early stage, the transcription levels of TNF-α, MCP-1 and IL-6 in mice. QRT-PCR was conducted to measure the gene expression levels of some typical inflammatory cytokines in the brains and lungs of EV-A71-infected and mock mice with TBK1 and IRF3 interventions at 1 dpi. Results were normalized by β-actin. Data represent mean ± SD. n = 3 per group; TBK1 and IRF3 interventions alone, EV-A71 *vs* Mock; TBK1 and IRF3 interventions with EV-A71 infection *vs* EV-A71; *P < 0.05; **P < 0.01; ns, no significant. All the experiments were repeated at least three times. The primers used in this experiment, IL-6: Forward: CTGCAAGAGACTTCCATCCAG, Reverse: AGTGGTATAGACAGGTCTGTTGG; MCP-1: Forward: TAAAAACCTGGATCGGAACCAAA, Reverse: GCATTAGCTTCAGATTTACGGGT; TNF-α: Forward: CCTGTAGCCCACGTCGTAG, Reverse: GGGAGTAGACAAGGTACAACCC.
(TIF)
