# Supplementary figures and images for: TBK1 and IRF3 are potential therapeutic targets in Enterovirus A71-associated diseases
Source: PLoS Negl Trop Dis. 2023 Jan 10;17(1):e0011001. doi: 10.1371/journal.pntd.0011001 (PMC9831319; doi:10.1371/journal.pntd.0011001)

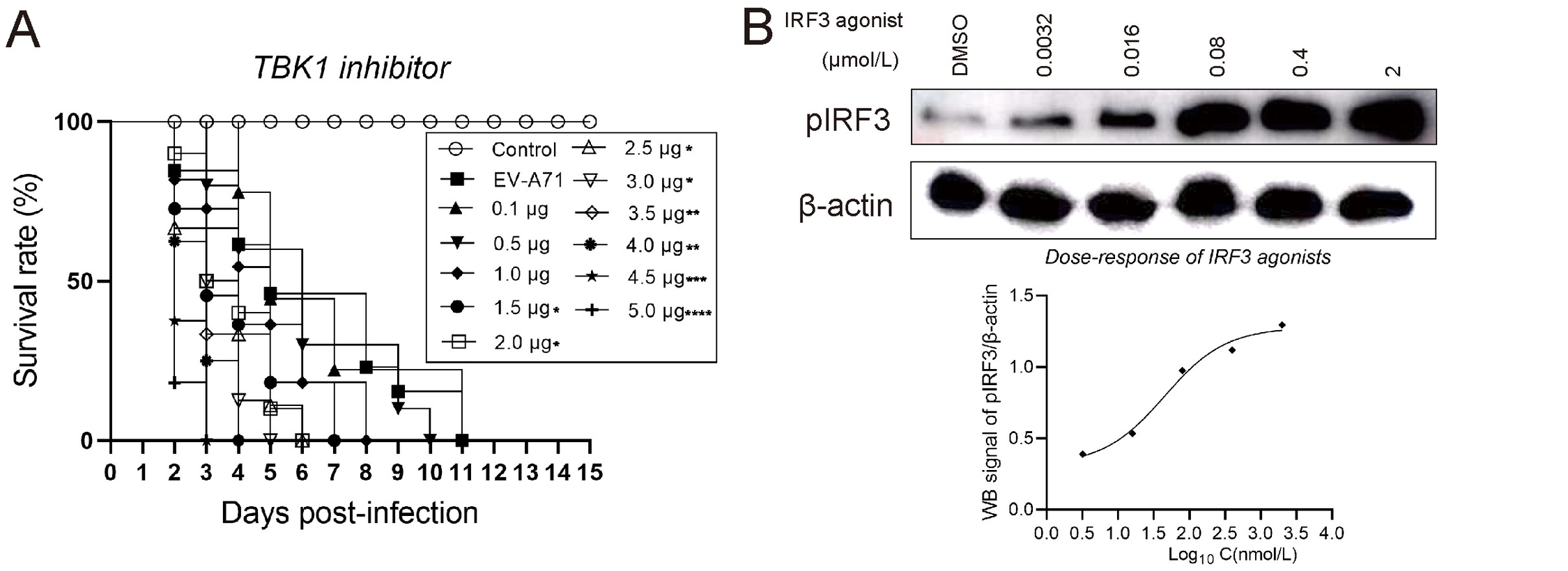

Supplement: S1 Fig — (A) 3-day-old BALB/c mice were inoculated intraperitoneally (i.p.) with 2.86 × 10^6^ TCID50 EV-A71 (ZZ1350 strain, OP806304), followed by i.p. injection with TBK1 inhibitor (dissolved in DMSO) to achieve a dose of 0.1–5.0 μg per mouse within 1 hour post infection (hpi). After being administered different doses of TBK1 inhibitor, survival rates of infected and control mice were recorded until 15 dpi. Finally, considering the survival rate, economic and reproducibility, 2.5 μg per mouse was selected as the appropriate intervention dose for following experiments. Statistical differences of survival rates between control and treated mice were assessed with the Mantel-Cox log rank test and survival curves were plotted using the Kaplan-Meier method. n = 10 per group; * P< 0.05; ** P< 0.01; *** P< 0.001; **** P< 0.0001. (B) IRF3 agonists treatment was dose-dependent with phosphorylated IRF3 (pIRF3) in vitro. (TIF) [file pntd.0011001.s001.tif]

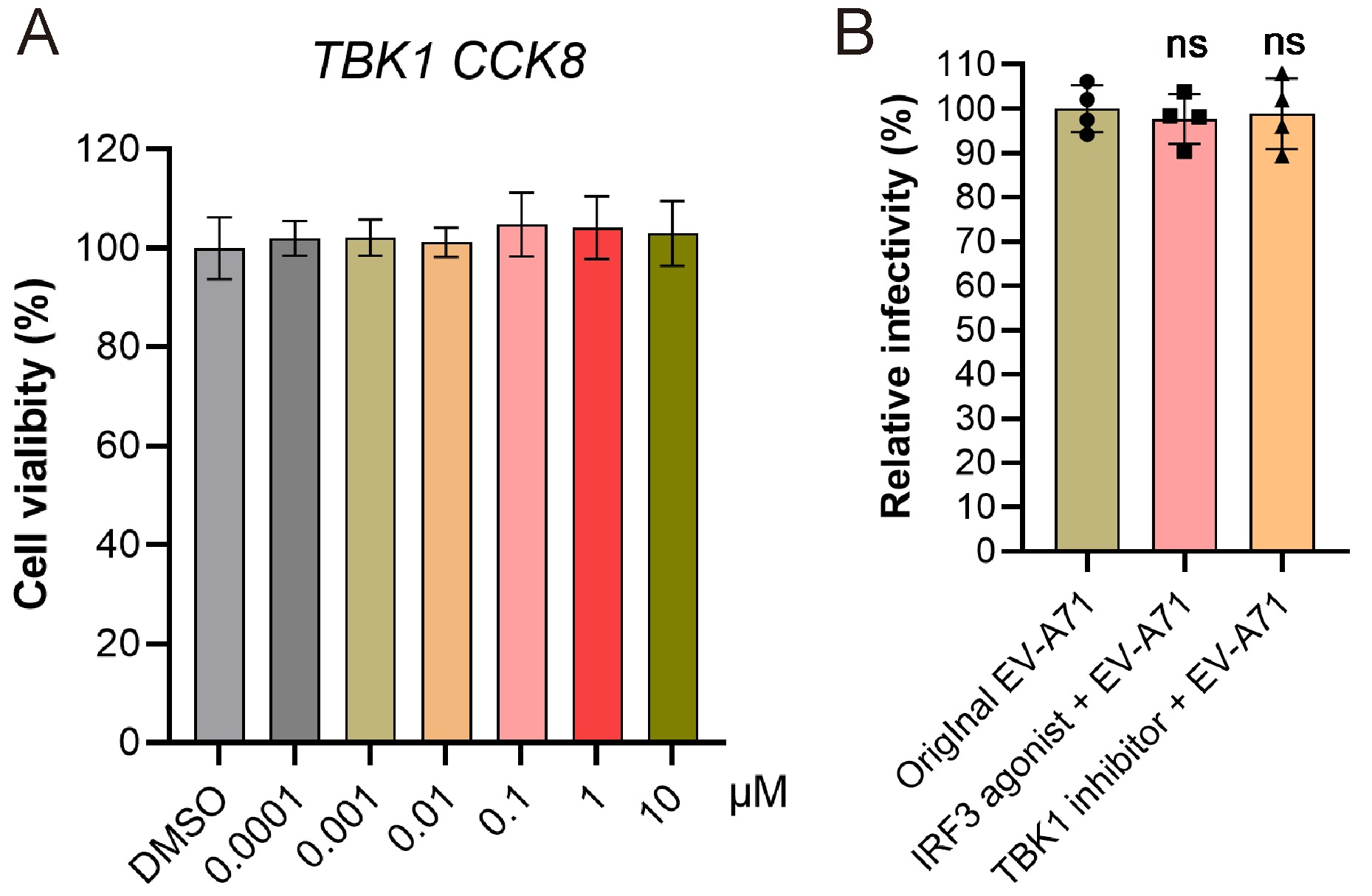

Supplement: S2 Fig — (A) 104 RD cells were seeded to each well of the 96-well plate and cultured for 24 h. Then, cells were cultured with DMSO or TBK1 inhibitor (0–10 μM) for 24 h before performing CCK8 assays (Biosharp, Cat#: BS350B)) according to the supplier’s instructions. n = 6 wells/per group (B) The agonist/inhibitor are not directly viricidal. The virus (2.86 × 10^8^ TCID50/mL) with or without agonist/inhibitor (100μM) was mixed and incubated for 1h at 37°C. We incubated the virus or the virus-agent mixture (MOI = 1) with monolayer RD cells for 1h at 4 °C (n = 4 wells/per group). After removing the culture medium, the cells were washed three times with cold PBS to remove the unbound viral particles. At 12 hpi, we freeze-thawed RD cells three times to assay the TCID50 of RD cell lysates and quantify the effect of the agonist/inhibitor on EV-A71 infectivity. Results were normalized by the infectivity of the original EV-A71. Statistical analysis was carried out using two-tailed unpaired Student’s t test. data represent the mean ± SD, ns, no significant. (TIF) [file pntd.0011001.s002.tif]

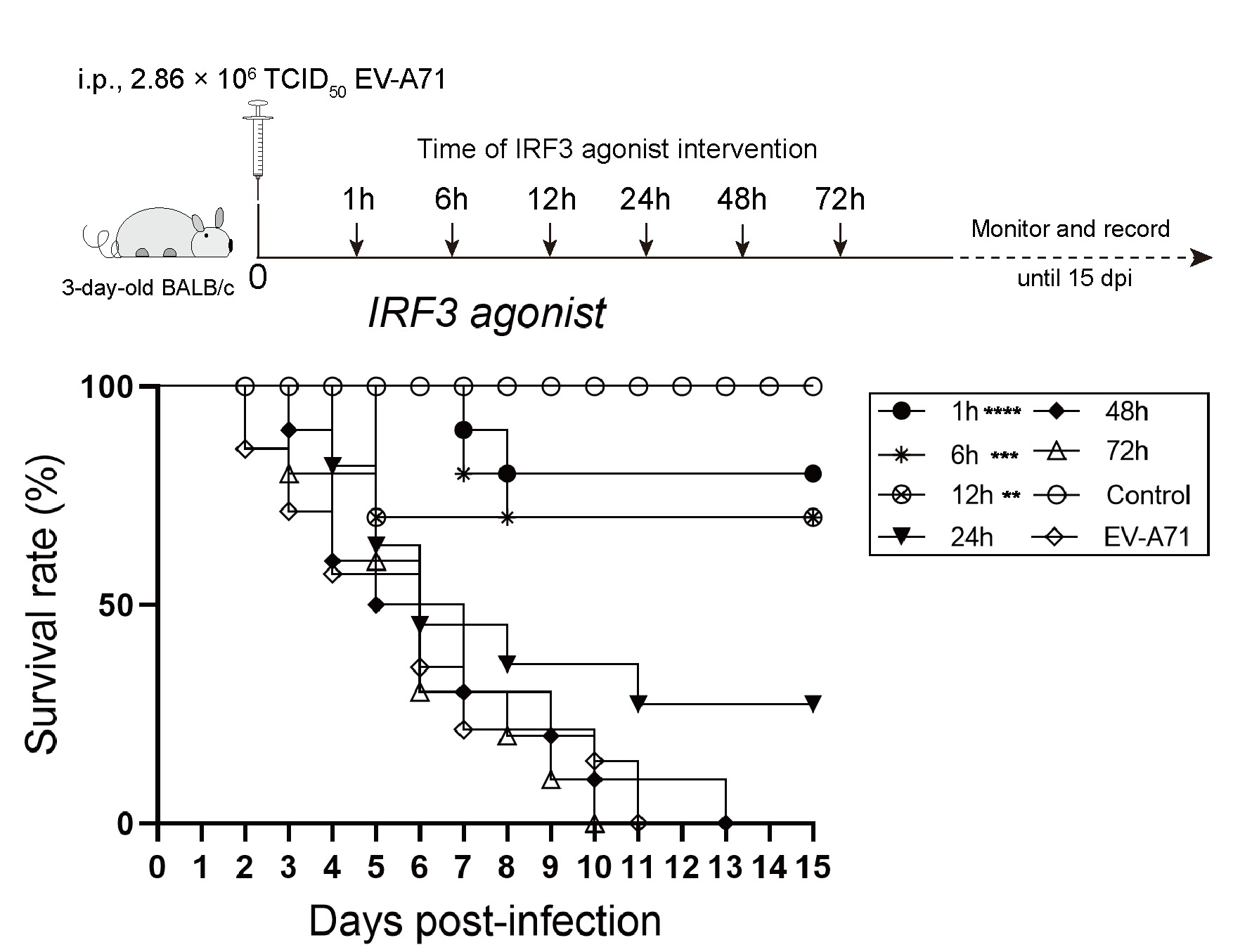

Supplement: S3 Fig — 3-day-old BALB/c mice were inoculated i.p. with 2.86 × 10^6^ TCID50 EV-A71 to infected the mice, followed by i.p. injection with 2.0 μg IRF3 agonist per mouse (dissolved in DMSO) at different time points (1, 6, 12, 24, 48, 72 hpi). Survival rates of infected and control mice were recorded until 15 dpi. Statistical differences of survival rates between control and treated mice were assessed with the Mantel-Cox log rank test and survival curves were plotted using the Kaplan-Meier method. n = 10 per group; ** P< 0.01; *** P< 0.001; **** P< 0.0001. (TIF) [file pntd.0011001.s003.tif]

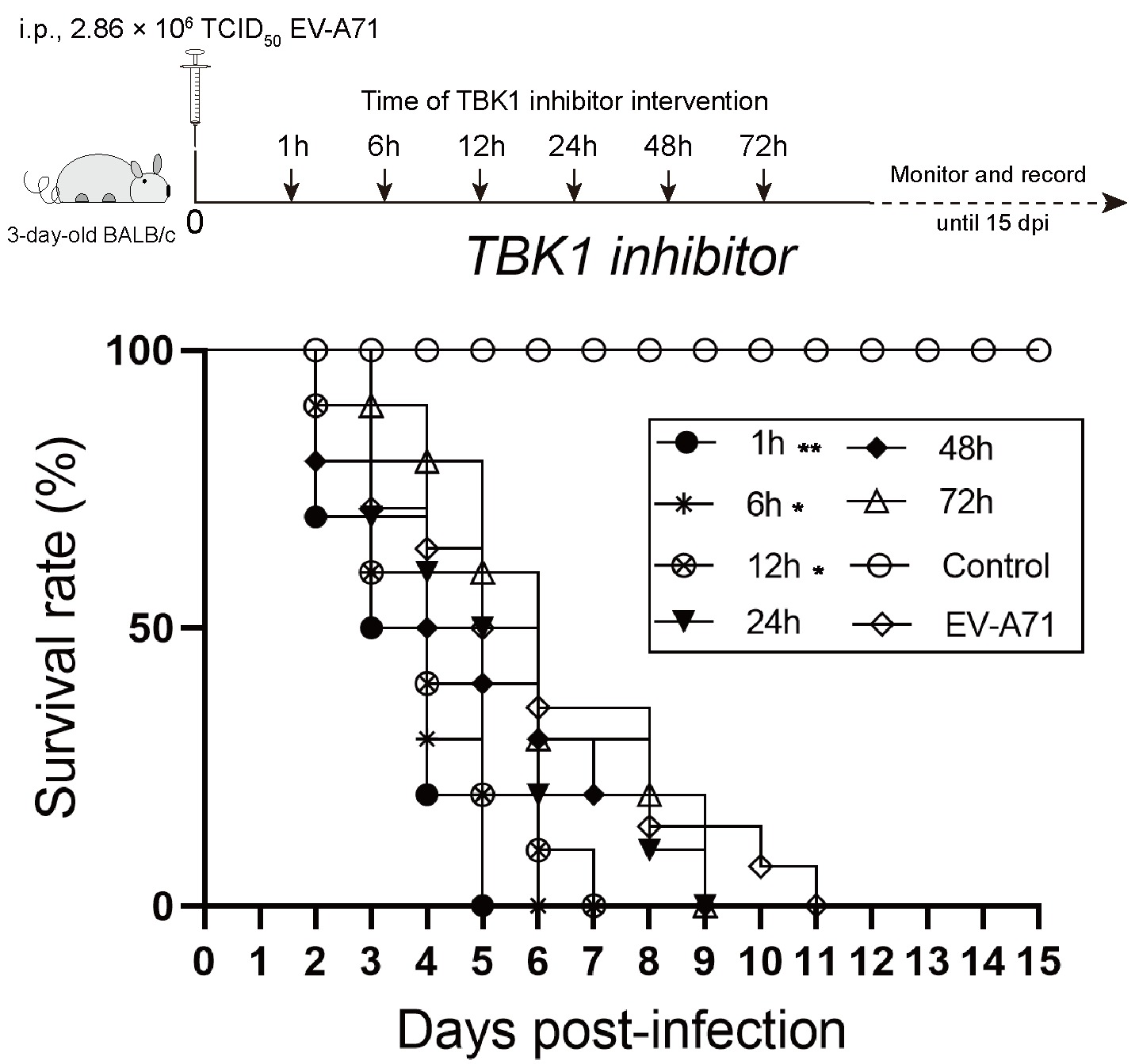

Supplement: S4 Fig — 3-day-old BALB/c mice were inoculated i.p. with 2.86 × 10^6^ TCID50 EV-A71, followed by i.p. injection with TBK1 inhibitor (dissolved in DMSO) to achieve a dose of 2.5 μg per mouse at different time points (1, 6, 12, 24, 48, 72 hpi). Survival rates of infected and control mice were record until 15 dpi. Statistical differences of survival rates between control and treated mice were assessed with the Mantel-Cox log rank test and survival curves were plotted using the Kaplan-Meier method. n = 10 per group; * P< 0.05; ** P< 0.01. (TIF) [file pntd.0011001.s004.tif]

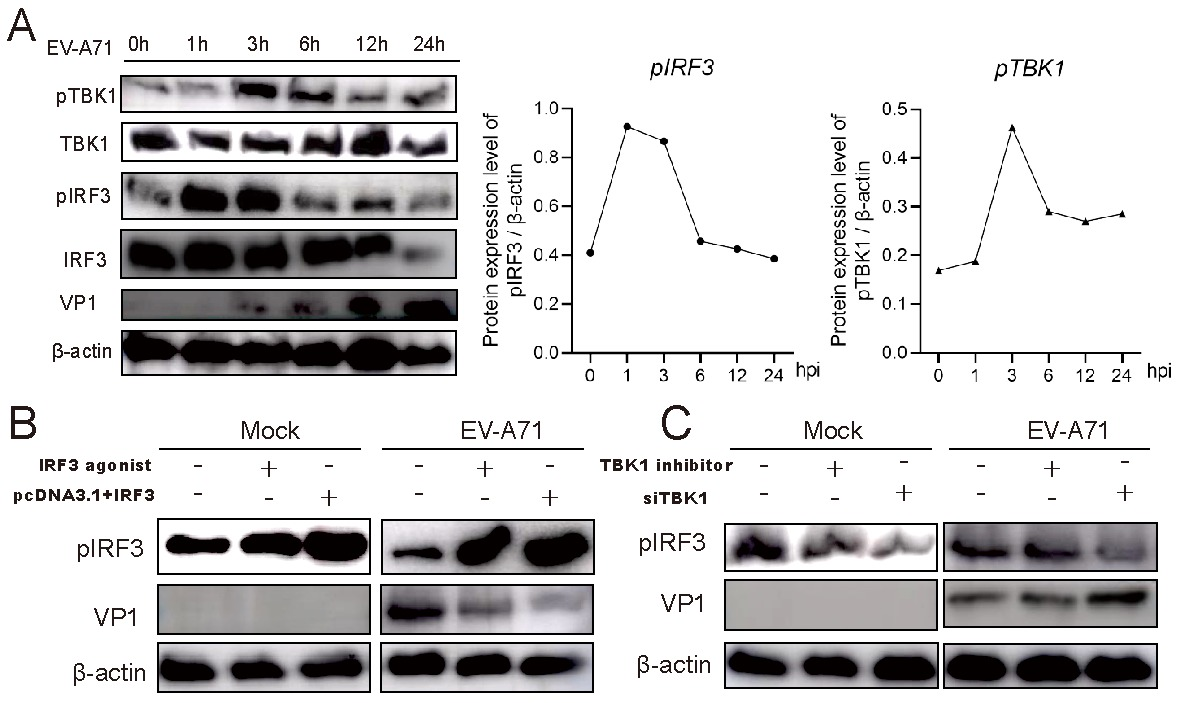

Supplement: S5 Fig — (A) Changes of phosphorylated TBK1 and IRF3 after EV-A71 infection, MOI = 1. (B) Overexpression of IRF3 (Synthesized by Shenzhen BGI Co., LTD) and IRF3 agonist (100 nmol/L) could decrease the VP1 expression of EV-A71. (C) siTBK1 (Guangzhou RiboBio Co., Ltd.) or TBK1 inhibitors (100 nmol/L) could increase the viral proliferation. The expression of related proteins was detected by WB analysis. (TIF) [file pntd.0011001.s005.tif]

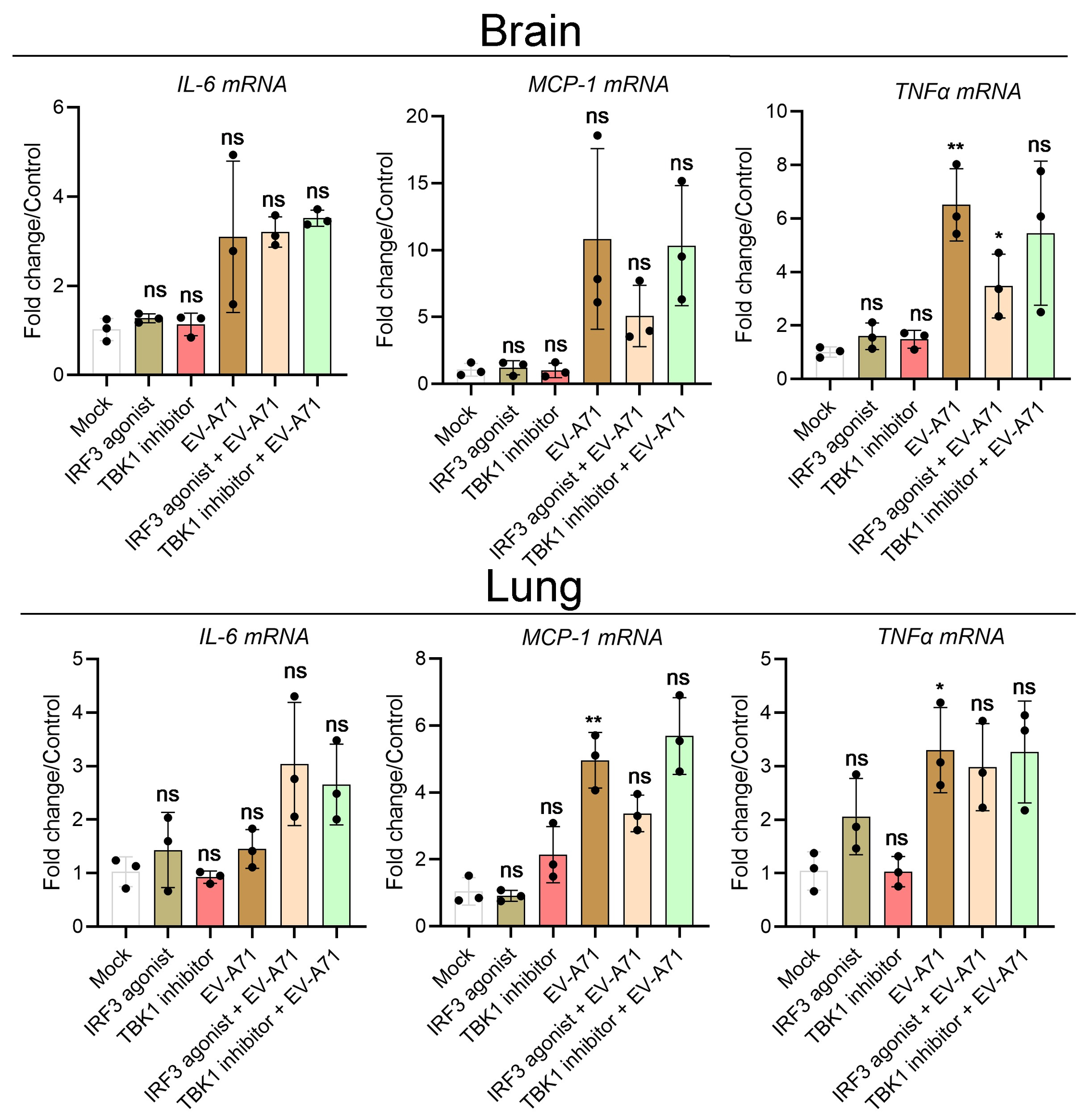

Supplement: S6 Fig — QRT-PCR was conducted to measure the gene expression levels of some typical inflammatory cytokines in the brains and lungs of EV-A71-infected and mock mice with TBK1 and IRF3 interventions at 1 dpi. Results were normalized by β-actin. Data represent mean ± SD. n = 3 per group; TBK1 and IRF3 interventions alone, EV-A71 vs Mock; TBK1 and IRF3 interventions with EV-A71 infection vs EV-A71; *P < 0.05; **P < 0.01; ns, no significant. All the experiments were repeated at least three times. The primers used in this experiment, IL-6: Forward: CTGCAAGAGACTTCCATCCAG, Reverse: AGTGGTATAGACAGGTCTGTTGG; MCP-1: Forward: TAAAAACCTGGATCGGAACCAAA, Reverse: GCATTAGCTTCAGATTTACGGGT; TNF-α: Forward: CCTGTAGCCCACGTCGTAG, Reverse: GGGAGTAGACAAGGTACAACCC. (TIF) [file pntd.0011001.s006.tif]
